# Image-Guided Percutaneous Drainage of Abdominal Abscesses in Pediatric Patients

**DOI:** 10.3390/children11030290

**Published:** 2024-02-29

**Authors:** Dimitrij Kuhelj, Crt Langel

**Affiliations:** 1 Institute of Radiology, University Medical Center Ljubljana, Zaloška 7, 1000 Ljubljana, Slovenia; 2 Faculty of Medicine, University of Ljubljana, Vrazov trg 2, 1000 Ljubljana, Slovenia

**Keywords:** percutaneous abscess drainage, appendicitis, children, pediatrics, Seldinger technique

## Abstract

Image-guided percutaneous abscess drainage (IPAD) is an effective, minimally invasive technique to manage infected abdominal fluid collections in children. It is the treatment of choice in cases where surgery is not immediately required due to another coexisting indication. The skills and equipment needed for this procedure are widely available. IPAD is typically guided by ultrasound, fluoroscopy, computed tomography, or a combination thereof. Abscesses in hard-to-reach locations can be drained by intercostal, transhepatic, transgluteal, transrectal, or transvaginal approaches. Pediatric IPAD has a success rate of over 80% and a low complication rate.

## 1. Introduction

Image-guided percutaneous abscess drainage (IPAD) is a minimally invasive technique that helps manage infectious complications by removing symptomatic or infected fluid collections. IPAD is the first-line treatment option for abdominal abscesses in pediatric patients in cases with no other concurrent indication for immediate surgery [1,2]. IPAD was first introduced in the late 1970s for adult patients but has since been adapted for children and is now frequently used in pediatrics [2,3,4]. The most common IPAD indication in children is an appendicular abscess [5].

In this paper, we review this abscess’ definition, pathogenesis, localization, presentation, imaging and diagnosis, treatment options, IPAD indications and contraindications, principles of catheter insertion and maintenance, and complications in the pediatric population. Additionally, we provide a review of the literature on IPAD.

## 2. Background

Abscesses are localized purulent fluid collections that can occur in all body locations accessible to bacteria, most commonly in or adjacent to the appendix or the sigmoid colon [6,7]. Abscesses arise when microbes breach a typically sterile area within the body, causing the immune system to respond by encapsulating the culprit germs, neutrophils, macrophages, exudative fluid, and necrotic cellular debris [8]. Initially, the purulent lesion is separated from the adjacent tissue by a pyogenic membrane. Granulation tissue then forms at the edge of the abscess. Two to three weeks after inoculation, the granulation tissue is replaced by a complete fibrous capsule containing pus, thus forming a chronic abscess [9,10].

In the general population, abdominal abscesses are often caused by appendicitis, diverticulitis, biliary disorders, pancreatitis, organ perforations, and penetrating abdominal trauma. Rarely, they develop as a result of bacteremia from a distant extra-abdominal focus [6]. In the pediatric population, the leading cause of abdominal abscesses is appendicitis [11]. Pediatric subgroups that may be especially susceptible to localized infections are newborns, oncologic patients, and immunodeficient patients [12,13]. The majority of abdominal abscesses are polymicrobial [14].

Abdominal abscesses may be classified based on their location, such as visceral (involving the liver, pancreas, spleen, kidneys, uterus, etc.) or non-visceral (including subphrenic, subhepatic, mesenteric, and paracolic abscesses) as well as intraperitoneal or retroperitoneal [15]. They can also be classified etiologically as inflammatory (resulting from conditions like cholecystitis, pancreatitis, appendicitis, and diverticulitis), trauma-related, or iatrogenic [16].

Small abscesses tend to have a benign course, provided they resolve while they are still small, either by means of antibiotic therapy or, rarely, spontaneous drainage [17]. However, abscesses may also be accompanied by systemic signs of infection, bacteremia, and sepsis. Sepsis often results in extended hospital stays and can give rise to multi-organ dysfunction [18].

There is a high variety in abdominal abscesses’ presentation, ranging from asymptomatic to full-blown systemic inflammatory response syndrome. Deep pelvic or retroperitoneal abscesses may be asymptomatic or present only as a fever, mild liver dysfunction, or ileus [19]. Shoulder tip pain or lung atelectasis are possible symptoms and signs of a subphrenic abscess [20]. The presence of a putrid odor, crepitus, hepatic portal venous gas formation, and necrotizing enteritis indicate anaerobic flora [21]. Owing to its unspecific presentation, abscess evaluation requires a careful correlation of clinical, laboratory, and imaging findings.

## 3. Imaging and Diagnosis

Imaging is crucial in abscess diagnosis and preprocedural planning, since it reveals the extent and anatomic relations of fluid collection and helps exclude other non-drainable pathologies, such as phlegmon. In babies and children, ultrasound (US) is preferred over CT primarily due to its absence of radiation exposure [22]. Also, diagnostics can be immediately followed by US-guided therapeutic procedures.

The choice of the most suitable diagnostic modality is based on clinical signs, children’s cooperability and age, the location of the abscess, the operator’s experience and preference, the availability of equipment, the ALARA principles (keeping radiation doses as low as reasonably achievable), costs, and other factors [23,24].

**Figure 1:**
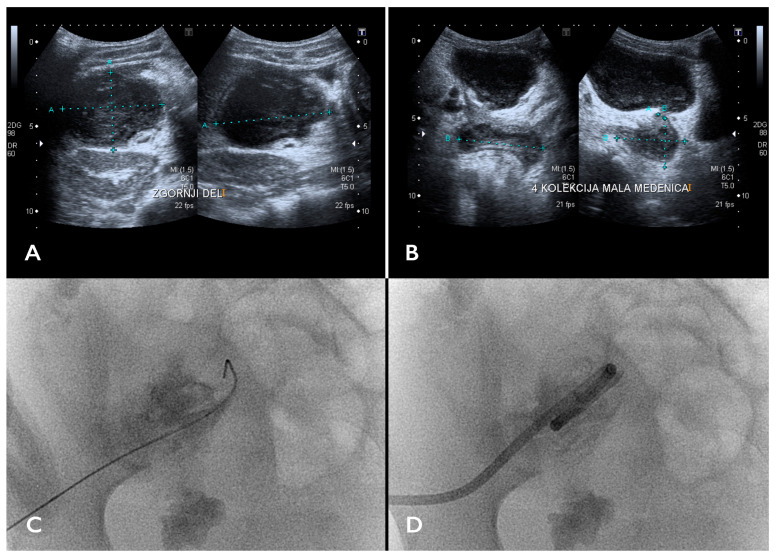
T (**A**) An ultrasound image showing an encapsulated fluid collection in the right hemiabdomen. (**B**) The caudal-most part of the multiloculated collection extended retrovesically. (**C**,**D**) Fluoroscopic image of image-guided percutaneous abscess drainage (IPAD) using a 10F drainage catheter.

Ultrasonographycally, abscesses typically appear as anechoic to hypoechoic, uni- or multilocular fluid collections with central layering debris, irregular ill-defined borders, or an echogenic capsule, with or without acoustic enhancement posteriorly, depending on fluid content (Figure 1A,B). Multiple echogenic foci and dirty shadowing may be seen, indicating gas within the fluid collection, which is, in the absence of a previous puncture or other air-introducing procedures, highly suggestive of an infection [25]. The presence of air bubbles helps confirm an abscess diagnosis, but the lack thereof does not rule it out [26,27]. Doppler US should be used to examine the fluid collections in the vicinity of vessels or inflamed pancreas to rule out pseudoaneurysms [28,29].

**Figure 2:**
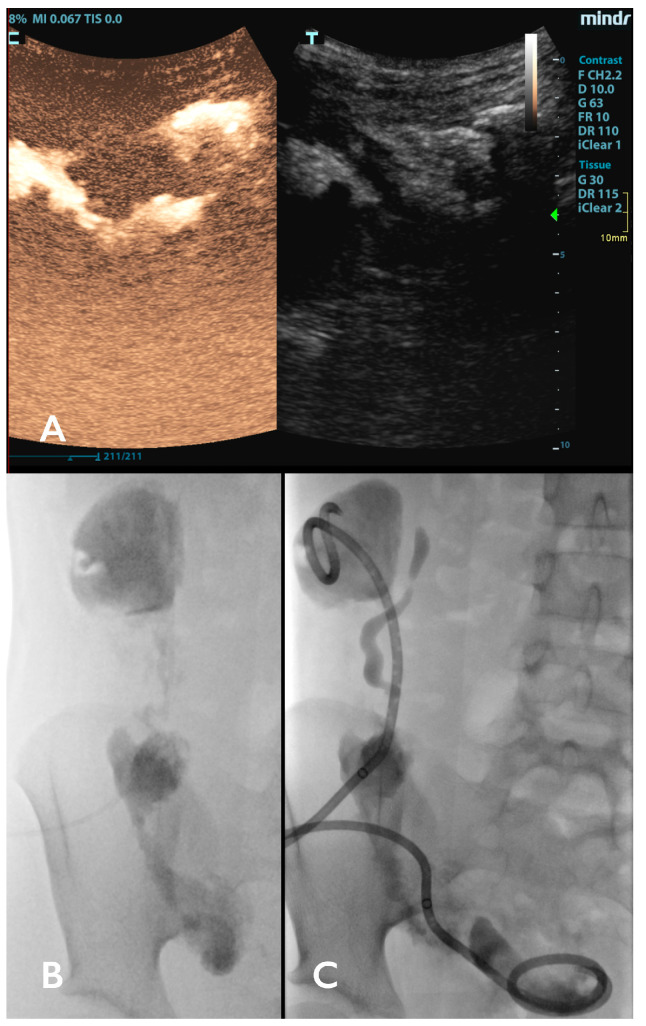
(**A**) One week after the IPAD, no clinical improvement was observed in the patient, and a follow-up contrast-enhanced ultrasound was performed injecting the SonoVue^®^ contrast directly into the abscess cavity using the inserted drainage catheter. CEUS revealed the communication between the iliac and paracolic abscess. (**B**) Another IPAD was immediately performed. The iodine contrast applied during fluoroscopy revealed three communicating abscess cavities that filled with the contrast applied under pressure, but the paracolic cavity then failed to drain upon iliac cavity drainage. (**C**) Then, a separate paracolic 10F catheter was inserted in addition to another new catheter, placed in the retrovesical abscess cavity. After ten days, the US follow-up revealed no signs of abscess (not depicted), and the patient was discharged.

On contrast-enhanced CT (CECT) (Figure 2), an abscess appears as a hypodense region, sometimes with even lower-density central part representing necrosis and a peripheral (rim) enhancement upon intravenous contrast administration [26]. There might be septations, loculations, or gas within the lesion [30]. The borders can be irregular and thicker compared to the thin smooth wall of a simple cyst. The surrounding tissues may show signs of inflammation (e.g., peritoneal fat stranding) or effacement due to the abscess’ mass effect [31]. Modern CT scanners can provide a fast diagnostic approach without requiring general anesthesia, especially in restless and agitated children, where adequate US examination may be difficult to perform. Also, not all centers provide 24/7 US diagnostics. In all such cases, the radiation burden and alternative diagnostic modalities should be taken into consideration.

Contrast-enhanced US (CEUS) is an advancement from traditional ultrasounds that is well suited for abdominal pathologies. It offers the benefits of ultrasound guidance along with real-time enhancement details and has minimal adverse effects [32]. CEUS helps identifying abscesses by increasing the tissue contrast between the non-enhancing abscess and the enhancing parenchymal organs, such as the liver, and by demonstrating the lack of vascularity within abscesses [33,34].

At times, even the specificity of CECT, US, and CEUS may not be sufficient to establish a clear diagnosis of an abscess. In such cases, fine-needle (22 to 20 gauge) diagnostic aspiration can be used to analyze the aspirate’s color, turbidity, viscosity, and odor. Fluid sampling is an excellent method to guide antibiotic therapy; Gram staining or antibiogram testing may also be performed. In smaller abscesses, diagnostic aspiration of no more than 5 mL of fluid is recommended to avoid collapsing the abscess cavity, thus rendering catheter insertion and end-loop formation difficult [35]. If no aspirate can be drawn despite an optimal needle tip position and up-sizing the needle to an 18 gauge, then performing a biopsy or placing a test drainage catheter are an option [2]. Especially in younger children, diagnostic drainage or puncture and drainage catheter placement should be performed under general anesthesia to reduce the pain burden, preferably in a single session to minimize anesthesia-related complications.

Fluoroscopy (Figure 1B–D and Figure 2C), apart from serving as an adjunct imaging modality in US-guided IPADs to confirm guidewire and catheter position, may also locate abscess complications such as fistulas [2]. Cone-beam computed tomography (CBCT) can provide an additional intra-procedural tool for navigating the pathways to the most difficult abscesses. However, the effective radiation doses in CBCT use are not neglectable, and its use should be limited to the most complex cases.

MRI, even without the administration of contrast media, is an excellent method for pathology detection, visualization, and safe access path planning [36]. Interventional MRI (iMRI) is also an established technique for IPAD, offering real-time three-dimensional needle guidance [2]. Nonetheless, the availability of dedicated iMRI suites and materials remains a challenge in the short term [2,37].

The main features of different imaging modalities are summarized in Table 1. 

## 4. Treatment

The typical treatment of an abdominal abscess involves a combination of antibiotics and percutaneous drainage or surgery. Combining medical therapy with invasive procedures is required as IV antibiotics generally do not reach effective concentrations within abscess cavities except in small immature abscesses [2,38]. Abscesses measuring 1–4 cm in diameter may occasionally resolve themselves by means of antibiotic treatment alone and, thus, warrant a wait-and-see approach [2,17,39]. A more aggressive approach should be based on the clinical course of disease. Non-resolving abscesses or children with systemic symptoms despite antibiotic therapy should be reassessed and often treated more invasively.

Historically, the go-to invasive approach for treating larger abdominal abscesses was surgical drainage and debridement, which carries considerable morbidity and mortality [40]. Since the late 1970s, IPAD has become the preferred primary invasive treatment option. 

## 5. Image-Guided Percutaneous Abscess Drainage

### 5.1. IPAD Basics

IPAD is a percutaneous “placement of a catheter with the use of image guidance to provide continuous drainage of a fluid collection”, as opposed to aspiration, which involves extracting the fluid and removing the needle or catheter immediately thereafter [1]. Unlike surgery, IPAD offers a simpler, minimally invasive alternative that tends to avoid general anesthesia, reduce iatrogenic trauma, prevent sepsis, shorten hospital stay, and lower the treatment costs [41]. In some cases, IPAD can be performed as an outpatient procedure [42]. The indications and contraindications for IPAD are summarized in Table 2.

### 5.2. Access Pathway

When choosing the access route to the fluid collection in the abscess, several considerations need to be made, including identifying the shortest and safest path to the abscess, ensuring optimal approach-path visualization, allowing for gravity drainage after insertion, determining the most comfortable and convenient location for the patient to manage the drain, and taking into account the preference and expertise of the operator [11,41,46,47]. Structures that should not be transversed with a needle include nerves, significant blood vessels, lungs, bowel, pancreas, spleen, kidneys, ureters, urinary bladder, and prostate [2,11]. Pleura, liver, and stomach may safely be transversed if needed [47]. To help create a larger window for catheter placement, hydrodissection can be used by injecting sterile saline along the access route [47]. In cases where no safe access can be found, alternative options such as antibiotic therapy alone or surgical treatment need to be considered [11].

Pediatric IPAD typically utilizes anterior and lateral abdominal transperitoneal approaches. The anterior approach is the most common and allows the patient to lie on their back during and after the procedure [48,49]. If an anterior approach is planned for an intra-abdominal collection, it is important to identify the location of the inferior epigastric arteries. Accessing through the skin just lateral to the linea alba can prevent injury to these vessels [47]. In the lateral approach, it is frequently necessary for the patient to remain in a decubitus or prone position, which can be less comfortable [26].

Accessing the posterior epigastric region can be challenging using an anterior approach due to the presence of the stomach, and it can be challenging when using a lateral approach due to the liver and the spleen. Additionally, in small children or infants, the lack of abdominal fat may further limit accessibility. In these instances, other approaches such as the posterior approach, lateral or medial to the kidneys, can be considered [2].

When a subcostal anterior approach is not feasible for accessing a subphrenic abscess, intercostal access may be necessary [21]. To ensure safety, the preferred approach is the most anterior and caudal approach possible because the anterior pleural reflection is situated farther cranially than the posterior one [2]. Intercostal access should be performed directly above the adjacent rib to avoid the neurovascular bundle that runs along the inferior surface of the upper rib [47]. Scalpel skin punctures to facilitate intercostal needle insertion should follow the same principle by pointing the blade’s sharp edge downwards. Although most patients do not experience pleural complications after undergoing intercostal subphrenic abscess drainage, close monitoring is essential for all patients [50]. It is recommended that ultrasound guidance be utilized to position a needle in the most caudal area of the fluid accumulation zone, with the needle tip pointing cranially, allowing the wire to migrate beneath the diaphragm. The tip of the catheter should then be placed just below the diaphragm for optimal subphrenic abscess drainage [2].

If standard access routes fail to treat abscesses within the lesser sac (omental bursa), an alternative transhepatic approach may be utilized that passes through the peripheral regions of the liver. However, it is advisable to steer clear of a more central transgression and use an 8 or 9F catheter to minimize the risk of damaging hepatic vessels or bile ducts [2]. Percutaneous transhepatic cholecystostomy catheter placement in children with acute acalculous cholecystitis has also been described, via Gelfoam-embolization of the non-mature puncture tract prior to catheter removal [51]. A transgastric catheter placement is also possible for pediatric abscesses in the lesser sac [2].

In children, deep pelvic abscesses can be accessed via transgluteal, transrectal, and transvaginal approaches. To perform a transgluteal placement, a catheter is inserted in close proximity to the sacrum, typically at the level of the sacrospinous ligament. The patient is positioned in a prone, prone oblique, or decubitus position. CT and CBCT guidance is usually preferred, but US guidance has also been described [52]. There is a risk of injuring important nerves and blood vessels in the greater sciatic foramen, so a puncture at the most posterior and inferior aspect of the foramen is preferred, ideally caudal to the pyriformis muscle to avoid the sacral plexus and reduce patient discomfort [50]. If the puncture must be located in the superior aspect, the needle should be as near to the sacrum as possible. Transgluteal access is a sterile procedure [26].

When abscesses are located near the rectum or posterior to the urinary bladder, a transrectal approach can be effective. The transrectal approach is generally well tolerated, with minimal complications, and allows patients to move around and defecate without difficulty [26]. Catheter dislodgement may occur, especially in the setting of constipation [53]. In smaller children, transabdominal rather than transrectal US imaging is usually preferred, visualizing needle and catheter advancement through the rectum into the abscess through a full bladder as an acoustic window [54,55]. The procedure is usually performed with the patient in a left decubitus or lithotomy position and is typically well tolerated because the rectum has limited pain receptors [53].

Transvaginal drainage is a viable approach for abscesses in the rectouterine pouch but is not commonly performed on pediatric patients due to the potential psychological impact. Employing endoluminal US guidance, it is better suited for adolescents who are familiar with tampon use or are sexually active [26]. Also not typically used in the pediatric population are the transperineal and transurethral approaches [2].

Finally, in post-operative abdominal abscesses that are not amenable to drainage by a simple over-the-wire exchange of surgical drains with radiologic drainage catheters, such as when the surgical drain is very long and tortuous or has already been removed, an alternative approach involves percutaneously puncturing the sinus tract created by a prior surgical drain. This method seems to reduce the risk of loss of the sinus tract (for example, due to its immaturity) and can be successful even when the skin opening of the sinus tract has healed [56].

### 5.3. Catheter Selection and Insertion

Catheters designed for drainage are typically made of a kink-resistant material, such as polyurethane, and may feature a hydrophilic coating to facilitate insertion. These catheters typically have multiple side holes positioned on the inside of the loop to increase flow. Although the achievement of successful drainage depends on several factors, the choice of catheter is a crucial factor to consider [41]. An 8F or 10F catheter is usually adequate for pediatric patients, while larger catheters are reserved for adolescent patients and collections which do not respond to initial drainage [26].

Two methods are available to an interventional radiologist for gaining access to the collection and securing a drainage catheter: the trocar and the Seldinger technique. In the trocar technique, the abscess is punctured under image guidance by a catheter mounted onto a sharp trocar needle, followed by catheter advancement, needle retraction, and catheter fixation [22].

The Seldinger technique involves using a needle to puncture the fluid collection, advancing a guide wire through the needle, removing the needle, using fascial dilators to create a track for the catheter, advancing the catheter over the wire to the depth of the collection, and removing the wire. A string-locking mechanism secures the catheter’s pigtail end in place. The catheter is then additionally secured to the patient with a skin suture or an adhesive-based locking device. The advantages and disadvantages of each method are summarized in Table 3.

In the Seldinger technique, wires may be advanced quite far into the abscess cavity to both break up potential loculations and offer ultrasonographic and/or fluoroscopic outlines of the abscess cavity by traveling along its inner wall. A sample of the fluids should preferably be aspirated and stored for further laboratory or microbiology evaluation before injecting any contrast material. Small volumes should be drawn to avoid abscess cavity collapse, hindering the subsequent catheter placement. Catheters should move over needles and wires with constant resistance; any rapid-onset reluctance or patient discomfort should raise suspicion of abscess cavity perforation. The final contrast examination of the cavity should be performed using no more than half of the aspirated volume, checking for possible fistula formation. The final drainage of the abscess cavity should only be performed after the drainage catheter is in situ and its pigtail, when present, locked [35].

### 5.4. Sedation and General Anesthesia

In children, sedation is often employed to relieve pain and anxiety and modify behavior to ensure the safe completion of a procedure. A child’s ability to cooperate during a procedure is dependent on their chronological age and cognitive development. Minor procedures like suturing small lacerations can be performed using distraction techniques, minimal sedation, and topical or local anesthetics. Involving children as “therapy partners” with detailed explanation of the procedure may decrease the use of sedation requirements. However, longer procedures that necessitate immobility in children under six years old or those with developmental disabilities generally require a deeper sedation to manage their behavior [44]. Sedation is a continuum with four recognized stages: minimal, moderate, and deep sedation and general anesthesia [57]. Sedation “may result in respiratory depression, laryngospasm, impaired airway patency, apnea, loss of the patient’s protective airway reflexes, and cardiovascular instability [57]”. Research indicates that children often slip into a deeper level of sedation than intended [54]. There may also be a tendency of the vast majority (about 90%) of both sedating and procedural physicians to underestimate the maximum depth of sedation, as indicated by a small single-center tertiary pediatric emergency department paper studying 50 children [58]. These findings underscore the critical importance of practitioners’ ability to rescue patients from deeper levels of sedation than initially intended for the procedure [57]. At our institution, minimal anesthesia may be performed by appropriately trained interventional radiologists, while deeper levels of sedation are performed by anesthesiologists. As a tertiary hospital, we have an anesthesiology team on site 24/7, so even unplanned pediatric drainage procedures tend to have all levels of sedation handled by anesthesiology specialists.

### 5.5. Catheter Management

Regular assessment of the catheter’s position, output monitoring, and flushing of the catheter with at least 10 mL of sterile saline solution every 8–12 h is necessary to maintain luminal patency, especially when the content is dense [22,41]. For more complex collections, intracavitary tissue plasminogen activator (tPA) with a normal saline solution has been suggested to facilitate drainage; this therapy can be performed as needed or on a set regimen [59,60]. However, a recent study by Gibson et al. found no benefits of tPA over a regular saline flush in terms of shorter catheter dwell time, procedure time to discharge, or time to resolution [61].

If necessary, the drain can be repositioned or exchanged for a larger size over a guide wire [62]. An additional catheter insertion may be needed to completely drain the abscess.

### 5.6. Efficacy and Complications

The reported technical and clinical success rates for adult IPAD range from 85 to 90% and 81 to 100%, respectively [11]. Clinical success is usually defined as freedom from surgery and abscess recurrence in a certain time period after drainage. The literature on pediatric percutaneous drainage suggests similar outcomes, with complications occurring in less than 5% of cases and major complications in less than 1% [4,49,58]. Potential complications include bleeding and abscess cavity wall rupture due to over injection or excessive guide wire manipulation with resulting septicemia, fistula formation, and clogging. Mispositioned catheters can cause bowel obstruction, perforation, or sepsis [62].

### 5.7. Follow-Up Imaging

Depending on clinical improvement, many pediatric patients require no follow-up imaging. Removal of the catheter depends on draining duration: generally, a week is enough to drain the majority of collections. US control can exclude the presence of remaining fluid and catheter displacement or blockage. Cases of prolonged stagnation, worsening, or complications warrant further imaging. At our institution, US follow-up performed by sub-specialized pediatric radiologists is primarily utilized to evaluate drainage adequacy, catheter position, and possible new abscesses’ formation. We also use CEUS when applicable. More complex cases or inconclusive US follow-ups are imaged using CT, with the incorporation of all dosage reduction measures. Postponed MRI, CT, or fluoroscopy imaging of suspected fistulae or other complications as well as additional pathology may also be performed, using the inserted drainage catheter for contrast injection.

## 6. Review of the Literature

PubMed was assessed for the purpose of collecting research data for inclusion in this review. A search was performed using the keywords “abscess” and “drainage” in combination with “children” or “pediatric”. The term abscess was intentionally not anatomically subcategorized to include as wide an array of abdominal abscesses as possible, e.g., hepatic, renal, or pelvic. The search results were evaluated for eligibility on the following criteria:

- clinical trial, randomized controlled trial, case series, or systematic review;
- a clear focus on intra-abdominal abscesses;
- a clear focus on percutaneous drainage in the pediatric population; one paper on abscess needle aspiration without drainage was also deemed appropriate for inclusion;
- studies focusing on surgical drainage or medical therapy were excluded;
- due to the fact that percutaneous drainage techniques have been well established for decades with little technical innovation and also considering the relative scarcity of published research, no time limit was applied during the literature search.

The literature search strategy yielded a total of 114 records. Titles, abstracts, and keywords were examined for their inclusion eligibility, and duplicates were removed, after which 11 records remained. The references cited by the included records and review papers yielded 5 additional inclusions, bringing the total to 16. The key findings of the published research on pediatric IPAD are summarized in Table 4.

The main takeaway from the published literature is that pediatric IPAD is safe and efficacious in treating abscesses in various locations, including in the pancreas, kidneys, and pelvis, as demonstrated by the overall high technical and clinical success rates and the low complication rate. The mean catheter dwell time ranged from 4 to 11 days. McCann JW et al. demonstrated the use of multiple catheter insertions in a single patient to be safe and efficacious [68]. McNeely et al. found that large and more diffuse abscesses significantly increase the rate of technical failure [69]. As mentioned, Gibson et al. showed that administering tPA via the drainage catheter did not show any advantage over regular saline flushing [61].

Highly variable rates of general anesthesia utilization were reported, ranging from 2% to 100% for technically similar procedures, indicating possible operator- or institution-related preferences [48,50].

Dotson et al. researched the variations in the management of abdominal abscesses in children with Crohn’s disease. They found out that, irrespective of the pediatric gastroenterologists’ experience level, the majority would choose CT for initial imaging (52%), followed by MRI (26%) and US (26%) [65]. On the other hand, they would prefer US imagining over other imaging modalities for follow-up imaging (47%) [65]. Another study by Dotson et al. found out that, at a single institution, CT was the imaging modality most frequently used for the follow-up imaging of Chron’s-related pediatric abdominal abscesses [66]. Such a practice differs quite significantly from that of our institution, favoring US for both initial and follow-up imaging, at least in uncomplicated cases. It is possible that these differences are simply due to a different availability of CT versus US and not due to different ALARA principles’ interpretation.

Linder et al. researched a small renal IPAD case series and found that the IPAD did not influence antibiotics’ selection, as the empirically administered antibiotic already matched the selection based on culture and sensitivity testing [67]. This possibly coincidental finding highlights the role of IPAD in the fine-tuning of antibiotic therapy in cases in which there, in fact, exists a mismatch between the empirically chosen antibiotics and those identified through culture-based methods.

In summary, further prospective pediatric IPAD studies with more rigorous initial and follow-up imaging modalities reporting and a standardized clinical success time window assessment could provide additional insights into this well-established treatment method.

## 7. Conclusions

IPAD is a simple yet essential procedure offering a safe and efficacious treatment for pediatric abdominal abscesses typically larger than 3 cm. It should also be considered in patients who remain febrile in spite of culture-specific medical therapy and in those who are immunocompromised or critically ill. Appropriately timed, it spares children the morbidity associated with surgery, making it a first-line treatment option. The technical and clinical success of this procedure depend on various factors, including knowledge of anatomy, abscess location, proficiency in imaging, appropriate approach selection and procedure planning, patient age, drainage technique, and adequate management of complications. The ALARA principles should be adhered to at all times, including during the initial and follow-up imaging.

## Figures and Tables

**Table 1:** Advantages and disadvantages of different abdominal abscess imaging modalities. Abbreviations: US—ultrasound; CECT—contrast-enhanced computed tomography; and MRI—magnetic resonance imaging.

	Advantages	Disadvantages
US	availability low cost no ionizing radiation real-time imaging of needle and catheter	lower specificity prone to image degradation operator dependent
CT	high specificity	ionizing radiation
MRI	high specificity	availability patient’s immobility, requiring anesthesia in children

**Table 2:** Image-guided percutaneous abscess drainage indications and contraindications.

IPAD Indications	IPAD Contraindications
- single uni-loculated abscesses at least 3 cm in size - multiloculated or multiple abscesses irrespective of size - abscesses with suspected enteric communication - abscesses smaller than 3 cm after failure of medical treatment - non-compliance with medical therapy - symptom relief (e.g., large collection causing pain, biliary tree, ureter, or bowel obstruction) [43,44,45]	- uncorrectable coagulopathy - lack of safe percutaneous access - lack of appropriate antibiotic prophylaxis - patient’s inability or unwillingness to cooperate - small immature abscesses - concomitant immediate surgery indication (e.g., peritonitis) [2]

**Table 3:** The advantages and disadvantages of the trocar and Seldinger catheter insertion methods.

	Advantages	Disadvantages
trocar	- simple - can be performed at the bedside	- requires a simple puncture trajectory - repositioning a catheter initially not inserted along the correct trajectory can be challenging
Seldinger	- can be used for harder-to-reach locations	- technically more challenging; deeper abscesses require fluoroscopy guidance - potentially more painful due to multiple steps

**Table 4:** Key findings of the published research on pediatric IPAD.

Study	Study Design	Aim	Results
Amundson GM et al., 1990 [63]	Retrospective study of four pediatric patients.	To assess the feasibility of the transgastric drainage of lesser sac abscesses following pancreatitis.	The approach is feasible. No major complications, minor complications: transient gastric venous bleeding, hematuria, and bleeding into a pseudocyst.
Burnweit et al., 1990 [64]	Retrospective study of 13 pediatric patients.	To assess the efficacy of the percutaneous drainage of traumatic pancreatic pseudocysts.	Six pseudocyst resolved spontaneously. Two were treated surgically. Five were treated by percutaneous drainage, with no complications or pseudocyst recurrence at the one-month follow-up.
Collins G et al., 2020 [39]	Retrospective study of 42 pediatric patients.	To assess the safety and efficacy of the non-operative management of small (<4 cm) post-appendectomy intra-abdominal abscesses.	Sixteen patients (38%) were treated with percutaneous drainage; twenty-six (62%) patients adopted non-operative management. In the drainage group, three patients required repeat percutaneous drainage and four required operative drainage. The non-operative management of post-appendectomy intra-abdominal abscesses is efficacious and safe.
Chung T et al., 1996 [50]	Retrospective study of seven pediatric patients.	To assess the safety and efficacy of the transrectal drainage (TRD) of deep pelvic abscesses using combined transrectal sonographic and fluoroscopic guidance.	Endovaginal US was used for initial catheter guidance, followed by fluoroscopy. General anesthesia was used in all cases. Mean catheter dwelling time: 4 days. 100% clinical success.
Dotson JL et al., 2013 [65]	Web-based survey of 248 pediatric gastroenterologists that were members of the North American Society for Pediatric Gastroenterology, Hepatology and Nutrition.	To assess the variation in the management of abdominal abscesses in children with Crohn’s disease.	Of the respondents, 52% would choose CT for initial imaging, 26% would choose MRI, and 21% would choose US. US would be preferred for follow-up imaging (47%), followed by MRI (33%) and CT (13%). Of the respondents, 77% would recommend percutaneous drainage as a first-line treatment and 21% as a step-up only after the failure of medical therapy. Only 2% of the respondents would recommend surgery as a first-line treatment. There were no clinically significant associations between treatment strategies and practitioners’ experience.
Dotson JL et al., 2015 [66]	Retrospective single-center study of 30 patients.	To determine the characteristics of the management of abdominal abscesses in children with Crohn’s disease in 28 patients who received either medical therapy or percutaneous drainage.	CT was the most common initial and follow-up imaging modality. The medical therapy group received significantly more follow-up CT imaging (67% v. 20%, *p* = 0.046). No significant differences were identified among the treatment groups for readmissions, complications, or abscess recurrence. After 1 year, 67% of the patients in the medical group and 60% of the patients in the percutaneous drainage group underwent surgery.
Gibson CR et al., 2021 [61]	Randomized controlled trial, with a sample size of 56 pediatric patients.	To evaluate the efficacy of once-per-day intracavitary tissue plasminogen activator (tPA) in the treatment of pediatric intra-abdominal abscesses.	Intracavitary tPA has no significant effect on the length of catheter dwell time, procedure time to discharge, or time to resolution.
Jamieson DH et al., 1997 [49]	Retrospective study of 46 pediatric patients.	To assess the clinical success rate and long-term (one-year follow-up) complications of simultaneous antibiotic and percutaneous drainage therapy of appendiceal abscesses.	Clinical success rate: 91%. Complications rate: 2%. Patients had more than one catheter inserted: 28%. Patients had additional catheters inserted in a separate session: 15%. Median catheter dwell time: 4 days.
Linder BJ et al., 2016 [67]	Retrospective study of three pediatric patients.	To assess the outcomes of pediatric patients with renal abscesses.	Indications for IPAD were the abscess size in two cases and the failure of medical treatment in one case. Clinical success rate: 100%.
McCann JW et al., 2008 [68]	Retrospective study of 42 pediatric patients with a total of 100 drainage catheters inserted.	To assess the safety and efficacy of multiple percutaneous drainages in children with acute complicated appendicitis.	Clinical success rate: 92.3%. Of the patients, 43% required reintervention (the other 56% presumably had more than one catheter inserted during the first session). Mean catheter dwell time: 8.2 days.
McNeeley MF et al., 2012 [69]	Retrospective study of 33 pediatric patients.	To evaluate the safety and efficacy of percutaneous drainage in children with perforated appendicitis.	Technical success rate: 87.9%. Appendectomy postponement rate: 100%. Large diffuse abscesses significantly increase the rate of technical failure.
Narang M et al., 2023 [70]	Randomized controlled trial with a sample size of 110 pediatric patients.	To evaluate the efficacy of ultrasound-guided needle aspiration in addition to antibiotics in children with uncomplicated liver abscesses.	Needle aspiration does not affect the clinical outcome at 6 weeks in children with uncomplicated liver abscesses. Needle aspiration may slightly reduce the duration of fever and abdominal pain/abdominal tenderness.
Pereira JK et al., 1996 [48]	Retrospective study of 45 pediatric patients.	To evaluate the efficacy of the transrectal drainage (TRD) and/or percutaneous drainage (PD) of deep pelvic abscesses.	All the patients recovered fully—both TRD and PD are effective in treating deep pelvic abscesses. Sedation was used in 44 procedures, while general anesthesia was used in 1 procedure. Mean catheter dwell times: 4.1 days (PD) and 5.5 days (TRD).
Rypens F et al., 2007 [71]	Retrospective study of 15 abscesses in 14 pediatric patients.	To evaluate the safety and efficacy of the percutaneous drainage of abdominal and pelvic abscesses in pediatric Crohn’s disease.	Complete abscess resolution in eight patients, partial in seven. One minor complication: an enterocutaneous fistula. Mean catheter dwell time: 11 days.
St Peter SD et al., 2015 [72]	Randomized controlled trial with a sample size of 62 pediatric patients.	To evaluate the efficacy of tPA irrigations after drain placement for appendicitis-associated abscesses.	The duration of hospitalization after drainage was significantly longer with the use of tPA. Medication charges were higher with tPA. There was no difference in the total duration of hospitalization, days of drainage, or days of antibiotics.
van Sonnenberg E, 1987 [4]	Retrospective study of 15 abdominal fluid collections.	To evaluate the safety and efficacy of percutaneous drainage.	Initial clinical success rate: 80%. Required surgery at a later time: 13%.

## Data Availability

Not applicable.
